# Fast and Large Shrinking of Thermoresponsive Hydrogels with Phase-Separated Structures

**DOI:** 10.3390/gels7010018

**Published:** 2021-02-16

**Authors:** Taehun Chung, Im Kyung Han, Jihoon Han, Kyojin Ahn, Youn Soo Kim

**Affiliations:** Department of Materials Science and Engineering, Pohang University of Science and Technology (POSTECH), 77 Cheongam-Ro, Nam-Gu, Pohang, Gyeongbuk 37673, Korea; thchung20@postech.ac.kr (T.C.); hik4251@postech.ac.kr (I.K.H.); lpchjh@postech.ac.kr (J.H.); ken784@postech.ac.kr (K.A.)

**Keywords:** hydrogels, thermoresponsive, poly(*N*-isopropylacrylamide) (PNIPAAm), phase separation, shrinking behavior

## Abstract

Thermoresponsive poly(*N*-isopropylacrylamide) (PNIPAAm) hydrogels have been attracting attention in a variety of functional materials, such as biomaterials, because they exhibit a volume phase transition phenomenon near physiological temperatures. However, the slow kinetics and small volume shrinkage of bulk PNIPAAm hydrogels upon heating greatly limit their practical application. Here, we report PNIPAAm hydrogels with phase-separated structures that exhibited ultrafast shrinking upon heating. The phase separation into a PNIPAAm-rich phase and a water-rich phase was formed through aqueous polymerization in the presence of NaClO_4_ salt. Through structural analysis of the hydrogels, a topologically heterogeneous and porous structure was observed, which was highly dependent on the NaClO_4_ concentration in the polymerization step. Compared to conventional PNIPAAm hydrogels, the phase-separated hydrogels exhibited much faster and larger shrinkage upon heating. Simultaneously, the hydrogels quickly released a large amount of water owing to the effective water channels inside them. The present method can be widely applied to general hydrogels, and it can address the numerous limitations of hydrogels in terms of operating programmability and deformation efficiency.

## 1. Introduction

When hydrophilic and stimuli-responsive polymers are three-dimensionally crosslinked and form hydrogels, this can change their volume in response to external stimuli. Poly(*N*-isopropylacrylamide) (PNIPAAm) hydrogels, the most investigated thermoresponsive hydrogels, exhibit sharp, reversible phase transitions in water at approximately 32 °C [1,2,3,4,5]. At this temperature, the so-called lower critical solution temperature (LCST), the volume of the hydrogels changes from a swollen state (below 32 °C) to a shrunken state (above 32 °C). This unique property of PNIPAAm hydrogels has been used in soft actuators [6,7,8,9] and biomedical applications [10,11,12].

One of the major drawbacks of PNIPAAm-based hydrogels is their slow deformation. In general, external stimuli are applied from the outside, and the hydrogel forms a hydrophobic skin layer on the surface, preventing water molecules from being released to the outside [13,14,15]. This skin layer is the main reason the volume shrinkage of the PNIPAAm hydrogels slows down upon heating. For example, Yoshida and coworkers reported that a PNIPAAm hydrogel disk with a thickness of 2 mm and a diameter of 15 mm takes 60 min to cause a 15% volumetric shrinkage [3]. To overcome the slow and small volume shrinkage of PNIPAAm hydrogels, comb-type grafted hydrogels [3,16] and porous hydrogels [17,18] have been explored. In particular, the macroporous structure formed by the phase separation during polymerization can accelerate the response rate upon heating because the water can diffuse out readily and rapidly during the shrinking process. Such phase-separated structures can be produced by synthesizing hydrogels at temperatures above the LCST [19,20,21] in aqueous solutions, mixed solvents [22,23], or NaCl aqueous solutions [18]. However, the above papers only compared the response speed; they did not specifically distinguish and analyze the PNIPAAm-rich and water-rich domains of the hydrogel matrix or calculate kinetics numerically. Moreover, in the case of studies using salts, only NaCl was reported, and the possibility of other salts was not explored.

It is known that Hofmeister ions, proposed in 1888 [24], are arranged in order of the ability of salts to precipitate certain proteins in aqueous solutions. Note that PNIPAAm also follows this trend associated with the LCST [25,26,27,28,29]. Specifically, as the concentration of Hofmeister anions increases, the LCST of PNIPAAm decreases, and the degree of the decrease differs depending on the type of anion. Based on this background, NaClO_4_, one of the Hofmeister anions showing a drastic effect on the LCST of PNIPAAm, was selected in this study. Using an aqueous NaClO_4_ solution as a reaction medium for carrying out the polymerization reaction, a series of PNIPAAm hydrogels with phase-separated structures were synthesized. As the NaClO_4_ concentration increased, the degree of phase separation increased and the void in the hydrogel matrix became larger. In addition, from confocal laser scanning microscopy (CLSM) and scanning electron microscopy (SEM) results, the hydrophobic domain in which the PNIPAAm was highly aggregated was clearly shown. By taking advantage of the preferential aggregation of PNIPAAm and the easy water transport, our hydrogels showed much faster and larger shrinking upon heating than conventional hydrogels.

## 2. Results and Discussion

### 2.1. Synthesis of Phase-Separated Hydrogels (PSGs)

**Figure 1:**
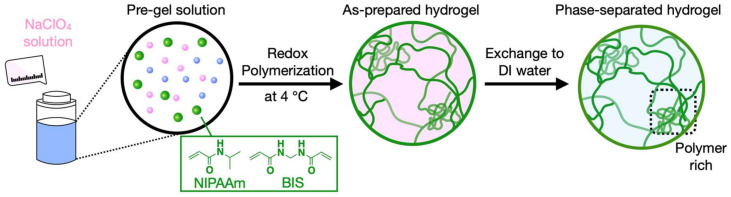
Schematic illustration of synthesis of phase-separated poly (*N*-isopropylacrylamide) (PNIPAAm) hydrogel (PSG).

Redox polymerization was performed in NaClO_4_ aqueous solutions by using ammonium persulfate (APS) and *N,N,N’,N’*-tetramethylethylenediamine (TEMED) as the initiator and the accelerator, respectively. A total of 1.6 M *N*-isopropylacrylamide (NIPAAm), 24 mM *N,N’*-methylenebisacrylamide (BIS), and NaClO_4_ with certain mass ratios were dissolved in 2.5 ml of DI water at 4 °C for each polymerization. For comparison, a normal hydrogel (NG) was polymerized in the same conditions without adding NaClO_4_. During the polymerization, the temperature was kept constant at 4 °C. The as-prepared hydrogels were washed twice a day for 4 d to remove residual NaClO_4_ and were stored in water at 20 °C for 3 d to achieve an equilibrium swollen state (Figure 1).

**Figure 2:**
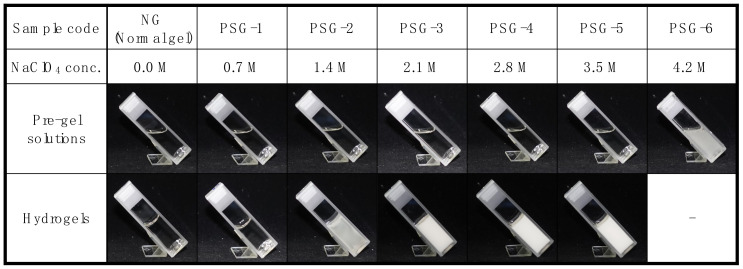
Photographs of pre-gel solutions and hydrogels in different conditions of polymerization.

The detailed sample code is as follows (Figure 2). The sample codes PSG-1 to PSG-6 were consistent with the concentrations of NaClO_4_ between 0.7 M and 4.2 M in steps of 0.7 M. In the case of 4.2 M NaClO_4_ of the pre-gel solution, monomers were not dissolved. In this study, we used 1.6 M of NIPAAm in the pre-gel solution, which was a relatively high concentration compared to the conventional PNIPAAm hydrogels. This was because when the concentration of NIPAAm in the reaction medium of PSGs was lower than 1.6 M, the resulting hydrogel easily crumbled or appeared as a viscous liquid.

According to the previous study reported by Sakota et al., the LCST of PNIPAAm in the 1.5 M NaClO_4_ aqueous solution was around 10 °C [26]. Increasing the concentration of ClO_4_^−^ lowered the LCST of PNIPAAm, so when the LCST dropped below 4 °C during polymerization, the growing PNIPAAm might have been aggregated, causing phase separation in the reaction medium. Indeed, the hydrogels polymerized in an aqueous solution of NaClO_4_ of 1.4 M or more began to become opaque, indicating the presence of phase-separated structures in the hydrogel matrix, as shown in Figure 2.

### 2.2. Characterization of PSGs 

**Figure 3:**
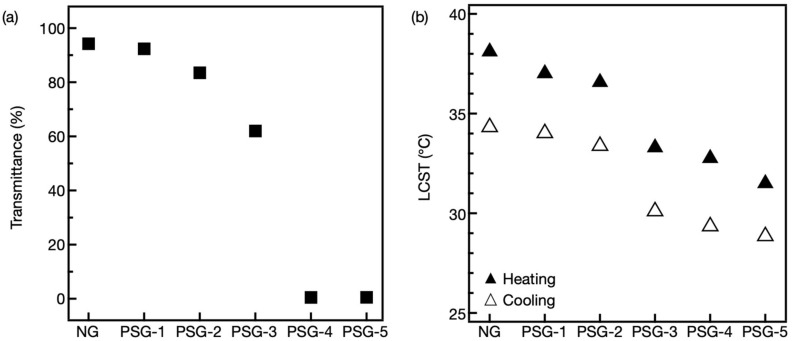
(**a**) Transmittance of NG and PSGs at 20 °C. (**b**) Lower critical solution temperature (LCST) of NG and PSGs upon heating and cooling observed by differential scanning calorimetry (DSC) (Heating, filled triangle; Cooling, open triangle).

First, the transmittance of the hydrogels was measured using UV transmittance at wavelengths from 600 nm to 700 nm (Figure 3a). At 20 °C, NG and PSG-1 were highly transparent, and the transmittance of each was more than 90%. As the NaClO_4_ concentration increased in the reaction medium, the transmittance of the hydrogels decreased gradually due to the high degree of light scattering of the phase-separated domains inside the hydrogels. In particular, from PSG-3 to PSG-4, the transmittance abruptly dropped from 61% to less than 1%. 

Next, differential scanning calorimetry (DSC) was used to investigate the LCST of the hydrogels. Prior to performing the experiments, the salts inside PSGs were removed through dialysis. The DSC thermogram of the hydrogels was analyzed from 15 °C to 55 °C (Figure S1), and the LCST of hydrogels was estimated through the one endothermic transition from the DSC curve (Figure 3b). Note that all hydrogels showed thermal hysteresis from the heating and cooling process, and the LCST was gradually decreased by increasing the NaClO_4_ concentration in the reaction medium. This phenomenon was presumed to be due to the small amount of NaClO_4_ remaining in the hydrophobic domains inside the PSGs.

### 2.3. Internal Structure of PSGs

#### 2.3.1. Observation of Hydrophobic Domains

**Figure 4:**
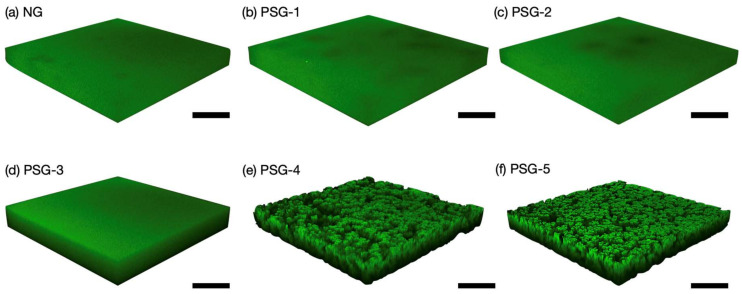
(**a**–**f**) Three-dimensional confocal laser scanning microscopy (CLSM) images of NG and PSGs at 20 °C. Scale bar, 50 µm.

The internal structures of the hydrogels were observed in the equilibrium swollen state at 20 °C using CLSM. CLSM systematically changes the distance between the lens and the sample, allowing cross-sectional images of varying depths to be observed. In addition, this series of cross-sectional images can be processed to generate three-dimensional images so that the three-dimensional internal structure of the sample can be grasped. No sample pretreatment such as drying is required, so it is convenient for “on-site” observation of the internal structure. Before the CLSM measurement, NG and PSGs were immersed at 20 °C for 3 d in an aqueous solution of 8-anilino-1-naphthalenesulfonic acid (ANSA), which is a fluorescent probe enabling the hydrophobic domain of hydrogels to be identified [30,31]. The specimen was sandwiched between the slide and the cover glass and then subjected to CLSM. From the CLSM images, NG, PSG-1, and PSG-2 showed homogeneous fluorescent intensity over all regions due to the absence of hydrophobic domains (Figure 4a–c). Although PSG-3 was an opaque hydrogel, it was difficult to detect the hydrophobic domains at the micron level, as shown in Figure 4d. On the other hand, the CLSM images of PSG-4 and PSG-5 obviously exhibited a large number of micron-sized hydrophobic domains (Figure 4e,f). The observed domain size was 15 µm and 50 µm in PSG-4 and PSG-5, respectively. These results indicated that micron-sized hydrophobic domains in the hydrogels were successfully formed when the concentration of NaClO_4_ was 2.8 M or higher, and they became denser and larger as the concentration was increased. The salt effect of lowering the LCST worked dramatically in PSG-4 and PSG-5, leading to phase separation into a PNIPAAm-rich phase and a water-rich phase during polymerization. Moreover, the resultant PNIPAAm-rich phase was successfully observed as the hydrophobic domains by CLSM.

#### 2.3.2. Observation of Morphologies inside Hydrogels

**Figure 5:**
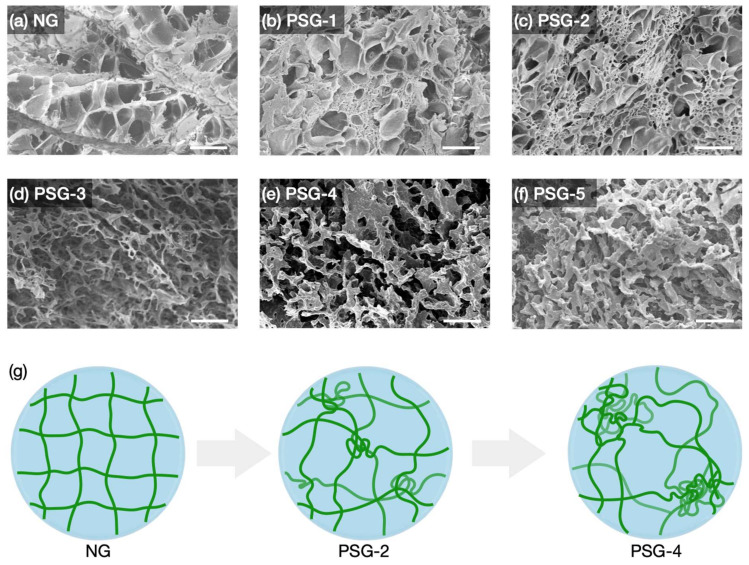
(**a**–**f**) SEM images of NG and PSGs. Scale bar, 10 µm. (**g**) Illustration of network structures according to degree of phase separation.

To visualize the morphologies of NG and PSGs using SEM, all the hydrogels were lyophilized. To lyophilize NG and PSGs, the swollen NG and PSGs were first immersed in liquid nitrogen, and the water inside them was quickly frozen into ice crystals. The frozen NG and PSGs were then dried by sublimating ice crystals under vacuum at a temperature below the freezing point of ice. For a homogeneous matrix of NG, the cross-linked polymer network in the matrix could be destroyed due to the expanded volume of water upon freezing [32]. Indeed, the pores of the lyophilized NG were roughly formed with a torn polymer network, as shown in Figure 5a. Therefore, the pores of the NG observed in the SEM images were created by ice crystals, not representing the actual mesh size in the swollen NG. 

In the SEM image of PSG-1, small and dense pores were observed, and their size as well as the occupied proportion was enlarged in PSG-2 (Figure 5b,c). Meanwhile, it appeared in a completely different form from PSG-3, where the polymer network was fused into thick and interconnected fibers, creating a huge void. From PSG-3 to PSG-5, the fiber thickness as well as the void size became larger (Figure 5d–f). Note that no destruction of the network was observed from the lyophilized PSG-3, PSG-4, and PSG-5. In other words, unlike NG, PSGs had separated domains between water and PNIPAAm at the micron level, allowing smooth sublimation without destroying the surrounding polymer (Figure 5g). Such massive voids observed in PSGs also provided an efficient water reservoir. The amount of water uptake of the polymer and the total water content in NG and PSGs were measured (Figure S2). As the NaClO_4_ concentration increased in the reaction medium, the amount of water that the polymer could uptake gradually increased. For example, PSG-5 could absorb 2.5 times more water than NG.

### 2.4. Thermoresponsiveness of PSGs 

The rod-shaped NG and PSGs with a diameter of 2.2 mm were equilibrated at 20 °C for 4 d. Then, the NG or PSGs were suddenly transferred into a hot water bath and held at 45 °C. As mentioned above, from the DSC results (Figure 3b), it can be seen that the LCST of the PSG was gradually decreased by increasing the NaClO_4_ concentration in the reaction medium by about 1 to 6 °C compared to NG. Since the temperature jump experiment was performed at 45 °C, which far exceeded the LCST of NG and PSGs, it was judged that the LCST of each hydrogel did not affect the degree and kinetics of shrinking.

**Figure 6:**
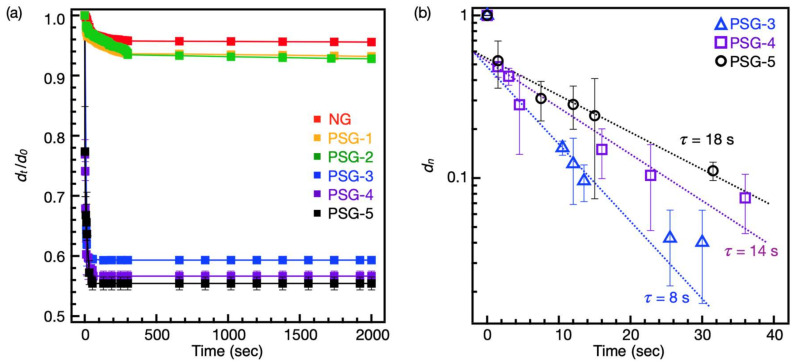
(**a**) Shrinking behaviors of NG, PSG-1, PSG-2, PSG-3, PSG-4, and PSG-5 after temperature jump from 20 °C to 45 °C, in which d_t_ was hydrogel diameter at time t and d_0_ was that of initial state. (NG, red; PSG-1, orange; PSG-2, green; PSG-3, blue; PSG-4, purple; PSG-5, black). (**b**) Plots of d_n_ against time. The relaxation times were determined from the slope (1/τ

At first, the initially transparent NG, PSG-1, and PSG-2 abruptly turned opaque, but detectable shrinkage hardly occurred. On the other hand, PSG-3, PSG-4, and PSG-5 were already opaque at 20 °C, so no further color changes were observed, but they abruptly shrank within 1 min at 45 °C (Figure S3). The shrinking behavior of NG and PSGs was investigated by measuring the change in diameter of the hydrogels after the abrupt temperature jump from 20 °C to 45 °C. Figure 6a shows the change in diameter (d_t_/d_0_) of the rod-shaped gel after the temperature jump to 45 °C, where d_t_ is the hydrogel diameter at time t and d_0_ is that of the initial state. NG, PSG-1, and PSG-2 slowly shrank, and the degree of shrinking was 4.5%, 6.9%, and 7.2%, respectively, after 2000 s. In fact, it took more than a month for these hydrogels to reach the equilibrium shrunken state [3]. On the other hand, in the case of PSG-3, PSG-4, and PSG-5, they abruptly shrank, and the degree of shrinking was 41%, 44%, and 45%, respectively, after 60 s. Notably, after 2000 s and even a month (data not shown), no more shrinking occurred, indicating that the equilibrium shrunken state had already been reached at 60 s. 

To investigate this phenomenon in detail, the shrinking kinetics were quantitatively analyzed. The kinetics of swelling gel were treated by Tanaka and Fillmore [33]. According to Equation (1), the relaxation time (τ) can be calculated from the slope of ($d_{n}$) versus time, where $d_{n}$, $d_{\infty }$, and τ are the normalized size, diameter in the equilibrium state at 45 °C, and relaxation time of the hydrogel, respectively.

(1) $$d_{n}\frac{\;d_{\infty }-d_{t}}{d_{\infty }-d_{0}}≅\;\;\frac{6}{\pi^{2}}e^{-\frac{t}{\tau }}$$

However, the above equation is applicable only when all of the following conditions are satisfied [34,35].

1. The time change in gel size (e.g., the gel diameter) can be described as a single-exponential function.
2. The hydrogel shrinks homogeneously without any surface skin layer formation, bubble formation, or wrinkling.
3. The uniform stress is applied to the gel in the initial condition, and the normal stress applied to the gel surface is zero in the boundary condition.
4. The time zone (t) used for the plot should be longer than the relaxation time (τ).

Therefore, the shrinking kinetics of conventional PNIPAAm hydrogels after a sudden temperature jump, in general, cannot be calculated using Equation (1) owing to heterogeneous shrinking or skin layer formation. Likewise, NG, PSG-1, and PSG-2 also exhibited heterogeneous shrinking, such as bubble formation or wrinkling, upon the temperature jump, as shown in Figure S3. In addition, the maximum time observed in this study was 2000 s, which was significantly shorter than the relaxation time of NG, PSG-1, and PSG-2, so the above equation could not be applied. 

Here, Equation (1) was applied to the shrinking process of PSG-3, PSG-4, and PSG-5 upon a temperature jump, since no heterogeneous deformation was observed in these hydrogels. As expected, the shrinking rate followed first-order kinetics, showing only one mode of relaxation. The relaxation times for PSG-3, PSG-4, and PSG-5 were 8, 14, and 18 s, respectively (Figure 6b). The reported relaxation times, calculated by temperature jump experiments using similar dimensions (several millimeters) of thermoresponsive hydrogels, were between 2410 and 6494 s. Therefore, it could be said that PSG-3 exhibited ultrafast shrinking kinetics that were about 300 times faster than those in previous studies [36,37].

Such fast and large shrinking of PSG-3, PSG-4, and PSG-5 could be explained as follows: the voids present in the hydrogel matrix provided an efficient channel for rapid water transport, resulting in the rapid response of hydrogels. Moreover, as observed in the CLSM images, PNIPAAm-rich and hydrophobic domains already formed within the hydrogel matrix could act as clusters to accelerate hydrophobic aggregation. Therefore, as these effects occurred comprehensively, PSG-3, PSG-4, and PSG-5 effectively squeezed out water, causing the large shrinking of the hydrogels.

) of the straight line (PSG-3, blue triangle; PSG-4, purple square; PSG-5, black circle).

## 3. Conclusions

We have established a simple and controllable method of synthesizing phase-separated PNIPAAm hydrogels through aqueous polymerization in the presence of NaClO_4_ salt. The LCST of the PNIPAAm effectively dropped at the optimized concentration of NaClO_4_ so that it afforded well-separated PNIPAAm-rich and water-rich domains at the micron level. Moreover, such phase-separated structures were identified through CLSM and SEM measurements. The resultant PSGs were opaque due to the high degree of light scattering of aggregated domains. Through the temperature jump experiment, PSG-3 showed the fastest and largest shrinking upon heating, with 45% shrinkage in 60 s as well as 8 s of relaxation time. These ultrafast thermoresponsive hydrogels can be applied in the field of actuators, solving the existing limitations of hydrogels in terms of operating programmability and deformation efficiency. Furthermore, they are expected to improve the efficiency of drug delivery systems by releasing large amounts of therapeutic agents in response to changes in temperature.

## 4. Materials and Methods

### 4.1. Materials

*N*-isopropylacrylamide (NIPAAm; Aldrich, St. Louis, MO, USA; 97%) was recrystallized from toluene/n-hexane. *N,N’*-methylenebisacrylamide (BIS; Aldrich, St. Louis, MO, USA; 99%), ammonium persulfate (APS; Aldrich, St. Louis, MO, USA; >99%), *N,N,N’,N’*-tetramethylethylenediamine (TEMED; TCI; >98%), and sodium perchlorate anhydrous (NaClO_4_, SAMCHUN; 98.5%) were used as received. As a dye for confocal microscopy images, 8-anilino-1-naphthalenesulfonic acid (ANSA; Aldrich, St. Louis, MO, USA) was used. DI water with resistivity 18.2 MΩcm (Direct-Q^®^ 5UV; Merck Millipore) was used throughout the experiments.

### 4.2. Synthesis of NG and PSGs

PSGs were prepared by redox polymerization. NIPAAm (1.6 M), BIS (24 mM), and NaClO_4_ with certain mass ratios were dissolved in 2.5 ml of DI water at 4 °C for each polymerization. PSG-1 to PSG-6 were synthesized with NaClO_4_ molar concentrations between 0.7 M and 4.2 M in steps of 0.7 M. Then, 30.42 µL of 1.3 M aqueous APS solution and 5.96 µL of TEMED were added into the system as an initiator and an accelerator, respectively, to initiate the redox polymerization, which was conducted at 4 °C for 1 h. Then, the as-prepared hydrogels were transferred to DI water at 20 °C, and the water was changed twice a day for 4 d to remove impurities. For comparison, NG was prepared in the same manner without adding NaClO_4_. Rod-shaped hydrogels with a diameter of 2.2 mm were prepared for the observation of shrinking, and disk-shaped hydrogels with a diameter of 18 mm and a thickness of 1 mm were prepared for the SEM and CLSM measurements.

### 4.3. Characterization of NG and PSGs

To measure the transmittance of hydrogels, we synthesized NG and PSGs in quartz cuvette cells and immersed them in DI water for 3 d to allow them to reach the equilibrium state. The transmittance was measured with a UV/Vis spectrometer (Lambda 750S, PerkinElmer) at a range of 200 nm to 800 nm. The final PSG transmittance was calculated to have an average value from 600 nm to 700 nm. 

Thermal transitions of NG and PSGs were measured using DSC (Q20, TA Instruments). The hydrogels in the equilibrium swollen state were taken for measurements. A thermogram for each hydrogel was obtained for the temperature range from 15 °C to 55 °C at heating/cooling rates of 5 °C/min.

The surface morphologies of NG and PSGs were observed using SEM. FE-SEM (S-4800, Hitachi) was used. The energy of the electron beam was 3.0 keV.

Confocal images were recorded on a CLSM (SP5, Leica) equipped with a UV laser (405 nm) and a 40 × water-immersion objective lens operating in fluorescence mode. Before the measurement, the hydrogels were immersed at 20 °C for 3 d in 0.05 wt% of ANSA aqueous solution. A three-dimensional view was constructed from sliced two-dimensional images using LAS AF software.

### 4.4. Shrinking Behaviors of Synthesized Hydrogels

The rod-shaped hydrogel samples were washed with excess DI water for 4 d, and the water was refreshed twice a day at 20 °C. The hydrogels were then quickly transferred into DI water at 45 °C, which was above their volume phase transition temperature. The hydrogels were observed using an optical microscope (SZX16, Olympus), and the images of the hydrogels were taken at specific times. The diameter of the hydrogels was analyzed using ImageJ software.

## Data Availability

Not applicable.

## Supplementary Materials

The following are available online at https://www.mdpi.com/2310-2861/7/1/18/s1, Figure S1: Differential scanning calorimetry (DSC) traces upon (a) heating and (b) cooling between 20 and 45 °C at a rate of 5.0 °C/min. Figure S2. Water uptake of polymer and water content of NG and PSGs. Figure S3. Shrinking behaviors of NG and PSGs. Optical images showing the (a) NG, (b) PSG-1, (c) PSG-2, (d) PSG-3, (e) PSG-4 and (f) PSG-5 after the temperature jumping from 20 °C to 45 °C. Scale bar, 1 mm.
