# Supplementary material for: Fast and Large Shrinking of Thermoresponsive Hydrogels with Phase-Separated Structures
Source: Gels. 2021 Feb 16;7(1):18. doi: 10.3390/gels7010018 (PMC7931089; doi:10.3390/gels7010018)
Supplement: Supplementary file 1 [file gels-07-00018-s001.pdf]

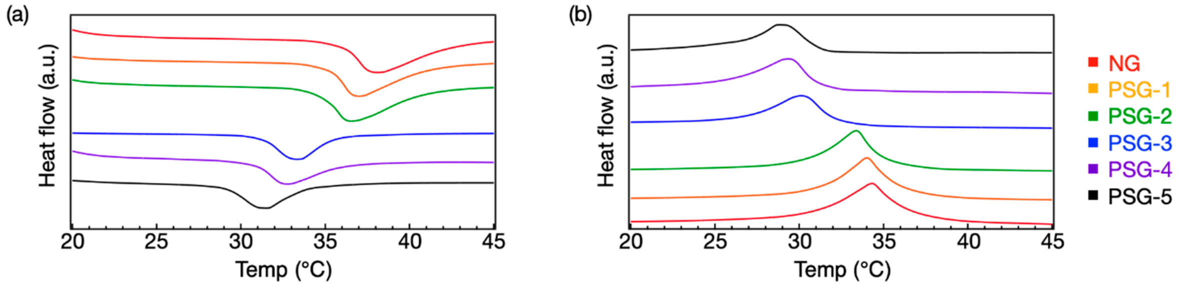

Figure S1. Phase transition behaviors of the hydrogels. Differential scanning calorimetry (DSC) traces upon (**a**) heating and (**b**) cooling between 20 and 45 °C at a rate of 5.0 °C/min.

Water uptake of polymer and water content in the hydrogels

The rod type of NG and PSGs was dried in vacuum until its weight was constant (W_d). The water uptake of polymer was calculated by the gravity method:

$$Water\ uptake\ of\ polymer = \frac{W_0 - W_d}{W_d}$$

where W_0 is the initial weight of the equilibrium state at 20 °C.

Also, the water content in NG or PSGs was calculated by the gravity method:

$$Water\ content\ in\ hydrogel = \frac{W_0 - W_d}{W_0} \times 100\%$$

where W_0 is the initial weight of the equilibrium state at 20 °C.

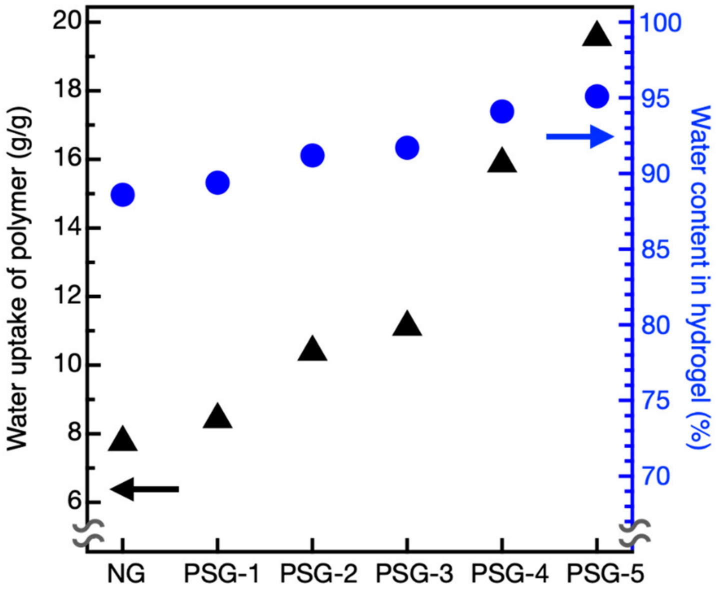

Figure S2. Water uptake of polymer and water content of NG and PSGs.

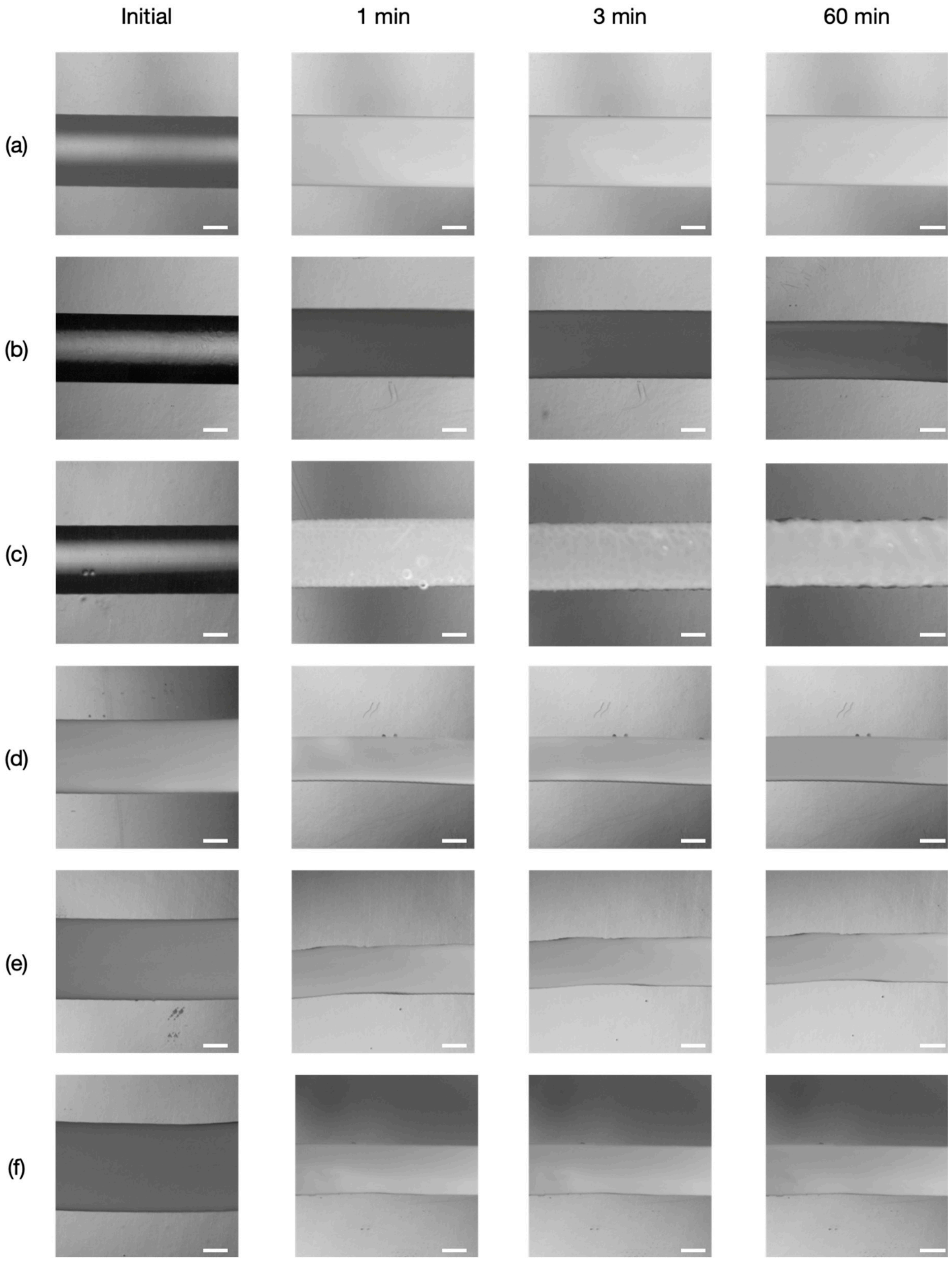

Figure S3. Shrinking behaviors of NG and PSGs. Optical images showing the (a) NG, (b) PSG-1, (c) PSG-2, (d) PSG-3, (e) PSG-4 and (f) PSG-5 after the temperature jumping from 20 °C to 45 °C. Scale bar, 1 mm.
